# Development of a Soft Robotic Bending Actuator Based on a Novel Sulfonated Polyvinyl Chloride–Phosphotungstic Acid Ionic Polymer–Metal Composite (IPMC) Membrane

**DOI:** 10.3390/membranes12070651

**Published:** 2022-06-25

**Authors:** Mohammad Luqman, Arfat Anis, Hamid M. Shaikh, Saeed M. Al-Zahrani, Mohammad Asif Alam

**Affiliations:** 1 Department of Chemical Engineering, College of Engineering, Taibah University, P.O. Box 83, Yanbu 41911, Saudi Arabia; 2 SABIC Polymer Research Centre, Department of Chemical Engineering, King Saud University, P.O. Box 800, Riyadh 11421, Saudi Arabia; hamshaikh@ksu.edu.sa (H.M.S.); szahrani@ksu.edu.sa (S.M.A.-Z.); 3 Center of Excellence for Research in Engineering Materials (CEREM), King Saud University, P.O. Box 800, Riyadh 11421, Saudi Arabia; moalam@ksu.edu.sa

**Keywords:** ionic polymer–metal composite (IPMC), sulfonated, polyvinyl chloride, phosphotungstic acid, platinum, membrane, robotic actuator, artificial muscle

## Abstract

This work presents the development of a cost-effective electric-stimulus-responsive bending actuator based on a sulfonated polyvinyl chloride (SPVC)–phosphotungstic acid (PTA) ionic polymer–metal composite (IPMC), using a simple solution-casting method followed by chemical reduction of platinum (Pt) ions as an electrode. The characterizations of the prepared IPMC were performed using Fourier-transform infrared (FTIR) spectroscopy, Scanning electron microscopy (SEM), X-ray diffraction (XRD) techniques, Thermogravimetric analysis (TGA), and Energy-dispersive X-ray (EDX) analysis. Excellent ion-exchange capacity (IEC) and proton conductivity (PC), with values of ca. 1.98 meq·g^−1^ and ca. 1.6 mS·cm^−1^, respectively, were observed. The water uptake (WU) and water loss (WL) capacities of the IPMC membranes were measured at 25 °C, and found to have maxima of ca. 48% for 10 h, and ca. 36% at 6 V DC for almost 9 min, respectively. To analyze the actuation performance of the developed membrane, tip displacement and actuation force measurements were conducted. Tip displacement was found to be ca. 15.1 mm, whereas bending actuation was found to be 0.242 mN at 4 V DC. The moderate water loss, good proton conductivity (PC), high thermal stability, and good electrochemical properties of the developed IPMC membrane actuator position it as a cost-effective alternative to highly expensive conventional perfluorinated polymer-based actuators.

## 1. Introduction

Recently, polymer-based actuators have come into existence because of their vital role in advanced materials and devices in various fields—including optics, electronics, and biomedicine, among others—as energy harvesters, micro-manipulators, biomimetic robots, etc. [1,2,3,4,5]. These materials can be considered soft actuating smart materials, usually made from electroactive polymers (EAPs) that show large strains under electrical stimuli, and respond quickly with large bending deformation. Polymer-based composite membranes are commonly classified into a few groups based on their characteristic properties: (a) elastic relaxation of shape after deformation; (b) changes in the orientation of mesogen groups; (c) reversible changes in the volume; and (d) those where the driving force is surface tension [6]. Recently, numerous articles have been published on EAP-based IPMCs—with interest focused on their cost-effectiveness, easy preparation, adaptability, light weight, and high performance—regularly referred to as ‘artificial muscles’, which have emerged as a potential precedent in naturally enlivened robots and biomedical frameworks [7,8,9,10].

Commonly, IPMCs consist of a thin ionomer or ion-exchange polymer membrane sandwiched between noble metal (e.g., Pt, Ag, Au) layers developed by electroless or chemical plating. In particular, IPMC-based membranes show a higher deformation than those based on other actuation materials, such as piezoelectrics [11]. A low voltage of 1-6 V DC can be applied for the membrane’s actuation, and can be used both in the air and underwater. IPMC-based actuators have some peculiar properties, such as being operable at low applied voltage, flexibility and light weight, easy molding into various shapes, good bending actuation, etc. Nevertheless, a time-consuming electroless plating method (a commonly used method) for the application of noble metal electrode sheets on both sides of the polymer membrane is usually essential for membrane fabrication of IPMC-based actuators. The development of electrochemical phenomena at the electrode surface is an important factor that facilitates the unique actuation process in the IPMC membrane [12,13,14]. For practical applications, IPMCs should have a tendency of large bending deformation under low applied electrode potential. The bending behavior of IPMCs depends upon a few basic properties of the IPMC membrane, including high IEC, high WU capacity, low WL capacity, and high PC (σ). In view of the characteristics of sulfonated polymeric membranes that maintain a balanced relationship between conductivity and thermal/mechanical stability, the modification processes—including hybridization, crosslinking, and grafting—have become efficient ways to sulfonate various types of homo- and copolymers [4,5,15,16,17,18,19].

Polymers based on perfluorosulfonic acid (PFSA)—e.g., Nafion^®^—possess great characteristics, such as high PC, marvelous stability against oxidation and chemical processes at moderate temperatures, and high performance; thus, these polymeric membranes are used for many advanced applications, including energy, separation, and actuation. However, the cost of perfluorinated polymeric membranes is usually very high, and its PC drops sharply with increasing temperatures. Due to these factors, however, there is a continuous search for cost- and performance-effective alternatives to these membranes, aiming to limit their large-scale use in practice [9,14,20,21,22,23].

In our recent works, we have successfully developed two different IPMC-based platinum-coated membranes—sulfonated polyether ether ketone–polyaniline and silicotungstic acid–sulfonated polyvinyl alcohol–polyaniline—showing good IECs, PCs, and actuation behaviors [5,14]. Various types of membranes with a combination of all or a few of these factors—including low-to-high performance, mechanical strength, flexibility, current density, tip displacement, actuation force, water absorption, loss capacities, etc.—need to be continuously developed for different soft robotic applications, as different applications may need different materials. Thus, the development of a polymer-based SPVC–PTA–Pt ionomeric membrane as a novel composite membrane is an effort in this direction. The main purpose of this study was to provide cost-effective IPMC membranes as an alternative to highly expensive commercially available membranes, e.g., Nafion. The prepared membranes were characterized using various techniques, such as FTIR spectroscopy, SEM, XRD techniques, TGA, and EDX analysis.

## 2. Materials and Methods

### 2.1. Materials

Polyvinyl chloride (PVC), phosphotungstic acid (PTA), 4-sulfophthalic acid [(HO_3_SC_6_H_3_-1,2-(CO_2_H)2-50 wt% aqueous solution), and hydrochloric acid (HCl-35%) from Sigma-Aldrich Chemie Pvt. Ltd., Burlington, MA, USA; ammonium hydroxide (NH_4_OH-25%) from Merck Specialties Pvt., Ltd., Darmstadt, Germany; and sodium borohydride (NaBH_4_) and tetraamine platinum(II) chloride monohydrate [Pt(NH_3_)_4_Cl_2_·H_2_O (Crystal form)] from Alfa Aesar, Tewksbury, MA, USA, were used in this study. The purchased analytical-grade chemicals and reagents were used in this experiment without further purification.

### 2.2. Instrumentation

An FTIR-ATR spectrometer (Thermo Scientific, Winsford, UK), XRD (Rigaku, miniflex-II, Tokyo, Japan), SEM (JEOL, JSM-6510 LV, Tokyo, Japan), potentiostat/galvanostat (PGSTAT 302 N autolab, Herisau, Switzerland), TGA/DTA recorder (DTG60H, Shimadzu, Tokyo, Japan), pH meter, magnetic stirrer, digital electronic balance, and electric air pump were used for processing and characterization of the prepared composite membrane.

### 2.3. Membrane Fabrication

**Figure 1:**
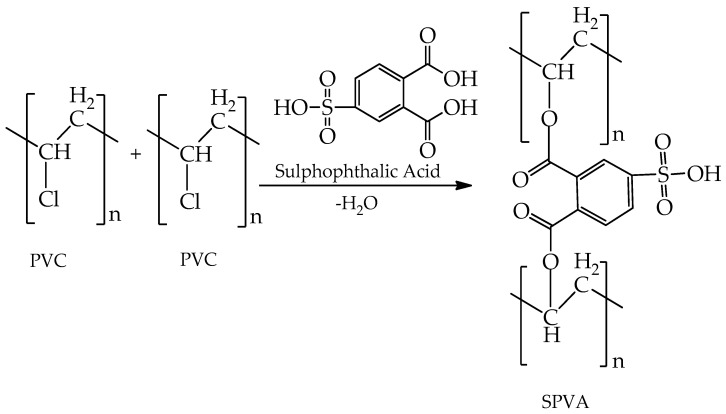
Scheme representing the mechanism of formation of the SPVC polymer membrane.

A polymer solution was prepared by dissolving 4.0 g of PVC polymer in 20.0 mL of demineralized water (DMW) at ca. 60 °C with constant stirring for 8 h for the fabrication of the membrane. After complete dissolution, 4.0 mL of 4-sulfophthalic acid was added to the solution for 4 h with constant stirring at the same temperature. Then, 1.0 g of PTA was added to the same solution for an hour with constant stirring at the same temperature and, finally, a homogeneous solution of SPVC–PTA polymer was prepared. The prepared solution was cast into a Petri dish (85 × 15 mm^2^) and covered with silver foil for slow evaporation, before being placed into an oven at ca. 45 °C for 12 h. A drying process was carried out, and then the SPVC–PTA polymer film was unstacked from the Petri dish and again placed in the oven for an hour at ca. 150 °C for self-crosslinking. Then, the fabricated membrane was ready for further study. Figure 1 describes the mechanism of the formation of SPVC.

### 2.4. Chemical Plating

Pt metal coating at the SPVC–PTA polymer surface was performed using an electroless plating method. The surface of the SPVC–PTA polymer membrane was roughed mildly from both sides with the help of sandpaper, and cleaning of the membrane was achieved using an ultrasonicator for 10 min. The membrane was then rinsed with HCl (2.0 M) at room temperature (25 ± 3 °C) for 8 h, before being neutralized with DMW. Next, 4.5 mL aqueous solutions of hydrated Pt(NH_3_)_4_Cl_2_ and 1.0 mL of /NH_4_OH were prepared and used to coat the Pt metal electrode on the SPVC–PTA polymer membrane. The membrane was placed for digestion in the reaction chamber at room temperature for 8 h. The prepared membrane was then immersed in DMW to release the excess Pt ions from the surface of the exposed membrane. About 5.0 mL of aqueous solution of NaBH_4_ was prepared and added to the Pt metal solution with constant stirring for ca. 1.5 h. Afterwards, the prepared membrane was cleaned with DMW and rinsed into a solution of 0.1 M HCl for the complete reduction of Pt ions into Pt metal. The prepared membrane was further kept for drying for 12 h.

### 2.5. Characterization of Actuator Membrane

The characteristic properties—such as IEC, PC, WU (by mass%), and WL (by mass%)—of the SPVC–PTA–Pt IPMC membrane were evaluated similarly as reported by Inamuddin et al. [24]. Structural features of the SPVC–PTA–Pt IPMC membrane were characterized using FTIR and XRD. Surface and cross-sectional morphologies of the studied IPMC membrane were investigated using SEM techniques, and their images were captured for observation for the coating of Pt on the surface of the polymer membrane. The thermal stability of the SPVC–PTA–Pt IPMC membrane was determined using a TGA analyzer at a constant rate of heating (10 °C·min^−1^) in a nitrogen atmosphere up to 800 °C. Electromechanical characterizations, including the maximum tip displacement and actuation force measurements of the examined IPMC membrane, were evaluated under a sinusoidal voltage of ±4 V.

### 2.6. Ion-Exchange Capacity (IEC)

The IEC (in meq·g^−1^), or the evaluation of the strength of H^+^ ions liberated from neutral salt moving via the polymer membrane, was determined using the standard column method. The dried SPVC–PTA IPMC membranes (0.25 g) were cut into small pieces and faded in HNO_3_ (1.0 M) for 24 h to convert into H^+^ form, and were then tested in neutral form with DMW, and placed in an oven for drying at 45 °C. The dried SPVC–PTA polymer membrane was now in a protonated form, which was compressed into a glass column. The eluent NaNO_3_ (1.0 M) was used to elute protons completely from the column, at an adequate flow rate of 0.5 mL·min^−1^. A standard solution of NaOH (0.1 M) was used to titrate the effluent by the use of an indicator (phenolphthalein), and the reported equation [24] was used to evaluate the IEC (meq·g^−1^) value.

The measured IEC value for the prepared IPMC was found to be 1.98 meq·g^−1^.

(1) $$\mathrm{Ion}\text{-}\mathrm{exchange}\;\mathrm{capacity}=\frac{\mathrm{Volume}\;\mathrm{of}\;\mathrm{NaOH}\;\mathrm{consumed}\;\times \;\mathrm{Molarity}\;\mathrm{of}\;\mathrm{NaOH}}{\mathrm{Weight}\;\mathrm{of}\;\mathrm{dry}\;\mathrm{film}}$$

### 2.7. Water Uptake (WU)

The WU capacity of the prepared membrane was examined at two different temperatures (25 ± 3 and 45 °C) for different intervals of time, i.e., 2, 4, 6, 8, 10, and 20 h. For this, the membrane was put into DMW for the corresponding times and temperatures. Thereafter, it was taken out of the water, and the surface of the membrane was cleaned with filter paper to remove the water droplets, after which it was weighed. WU capacity was evaluated with the help of the following equation [24]:

(2) $$\mathrm{WU}=\frac{W_{wet}-W_{dry}}{W_{dry}}$$

where $W_{wet}$ and $W_{dry}$ are the weights of the wet and dry membranes, respectively.

### 2.8. Water Loss (WL)

The IPMC membrane was first placed in DMW for 10 h at 45 °C for the maximum absorption of water. It was then taken out of the water and, after cleaning the water droplets from both surfaces, it was weighed. The WL capacity of the weighed IPMC membrane was evaluated through electric potential applied at a range of 3–6 V for short intervals of time (3–9 min). The WL of the IPMC membrane was evaluated using the following equation [25]:

(3) $$\%\;\mathrm{WL}=\frac{W_{1}-W_{2}}{W_{1}}\;\times \;100$$

where *W*_1_ and *W*_2_ are the weight of the hydrated membrane before and after the application of the electric potential, respectively.

### 2.9. Proton Conductivity (PC)

The PC of the hydrated IPMC membrane (1 × 3 cm^2^) was evaluated at 25 °C using an impedance analyzer that was connected with an Autolab 302N modular potentiostat/galvanostat, over a frequency of 100 kHz and an AC perturbation of 10 mV·s^−1^. The PC (σ) of the IPMC membrane was evaluated using the following formula [24]:

(4) $$\sigma =\frac{L}{R\times A}$$

where σ, *A*, *R*, and *L*, are the PC, cross-sectional area (cm^2^), resistance (ohms), and thickness (cm) of the studied IPMC membrane.

### 2.10. Electromechanical Study (ES)

**Figure 2:**
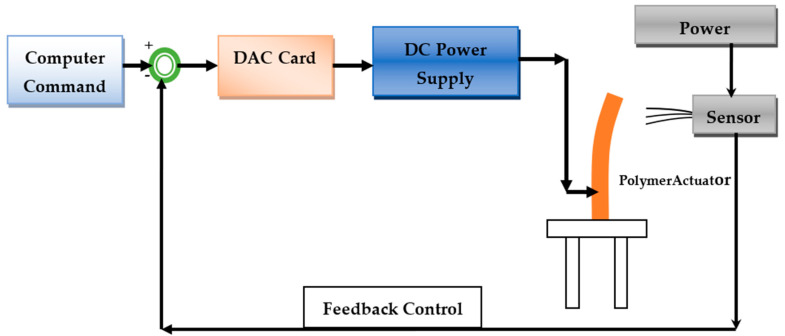
Basic layout for actuation and control of the SPVC−PTA−Pt polymer actuator.

In order to determine the electromechanical properties of the SPVC–PTA–Pt polymer soft actuator, a test setup was established, and the basic layout for the actuation and control of the SPVC–PTA–Pt polymer actuator is shown in Figure 2.

**Figure 3:**
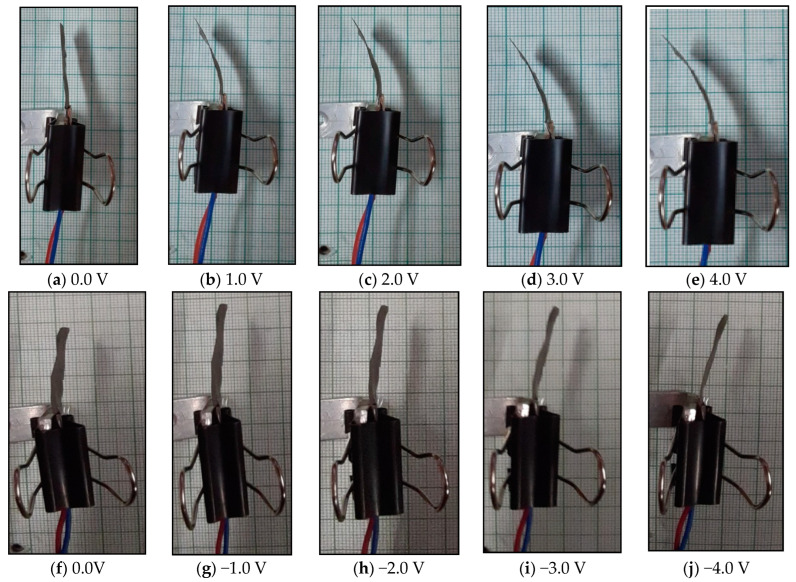
Step-by-step bidirectional deflection behavior of the SPVC–PTA–Pt actuator.

The polymer actuator membrane (ca. 30 mm × 10 mm × 0.2 mm) was fixed in a holder that was mounted in a cantilever mode. To supply the voltage to the actuator, a digital power supply and digital–analogue card (DAC) were used, where an electrical pulse was sent through the command control operation. For controlling the voltage, basic digital–analogue card software was used, where the input command was sent by providing voltage of up to 6 V DC. A laser-based displacement sensor was used to determine the tip displacement of the membrane after applying the potential. This provided feedback during the operation of the PVC–PTA–Pt polymer. The successive bending responses after applying different voltages are shown in Figure 3.

**Figure 4:**
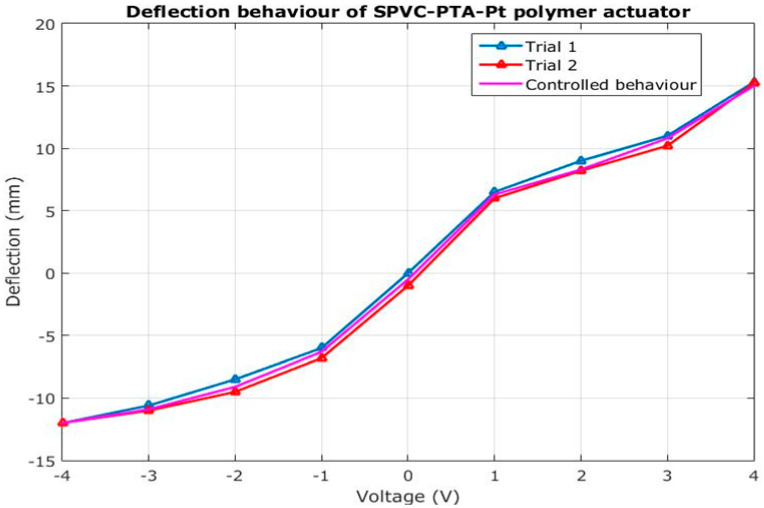
Deflection behavior of the SPVC–PTA–Pt polymer actuator obtained experimentally.

The tip displacement data of the actuator are given in Table 1. The bidirectional deflection behavior of the actuator is also plotted, as shown in Figure 4. It can be seen that the bidirectional deflection behavior of the actuator has some sort of hysteresis. The experiments were also repeated, and it was found that the hysteresis behavior showed that the tip deflection of the actuator increased during the increase in the voltage. The deflection error (i.e., hysteresis) was overcome by setting up the voltage in DAC while controlling the actuation process of the actuator. The deflection under positive voltage (0-positive 4 V DC) was slightly greater than that at the same negative voltage (0-negative 4 V DC), as upon applying the voltage, the behavior of the actuator was not very uniform, due to hysteresis. This was minimized by applying control systems. The grid size on the X-axis was 1 V DC, while that on the Y-axis was 5 mm deflection on both the positive and negative sides. The voltage cycle was repeated for 10 trials to determine the stable deflection. When the voltage cycle was repeated, the bending deflection of the actuator was stabilized.

## 3. Results and Discussion

### 3.1. IEC, PC, WU, and WL

**Figure 5:**
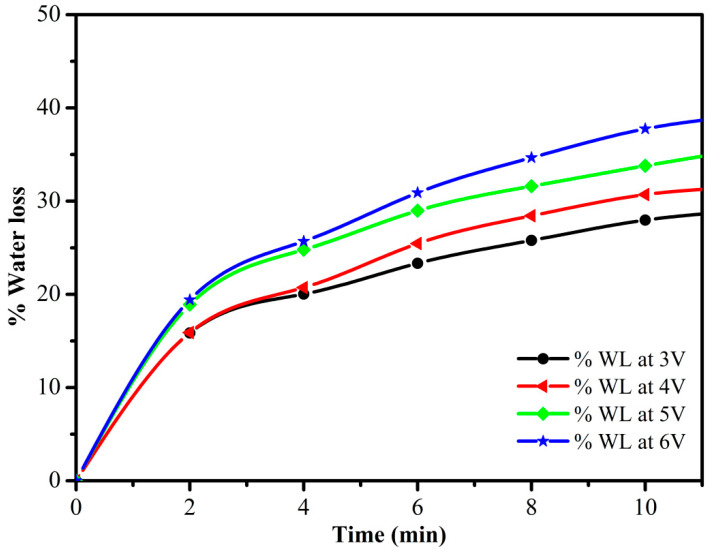
Water loss of the SPVC–PTA–Pt IPMC membrane at 3–6 V DC.

**Figure 6:**
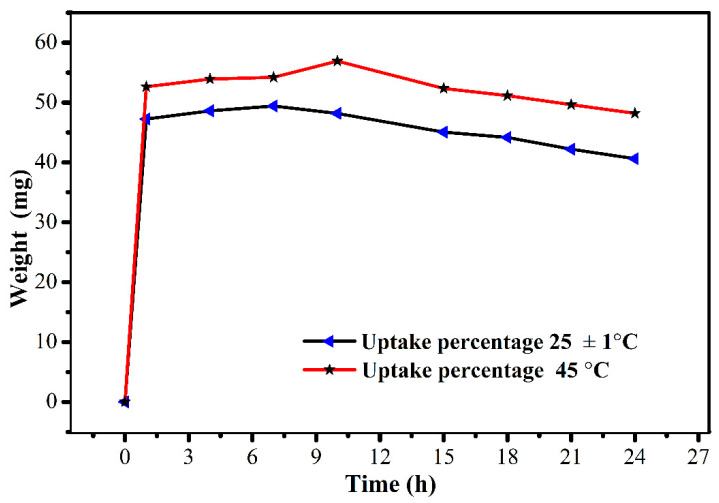
Water uptake of the SPVC–PTA–Pt membrane at room temperature (25 ± 3 °C) and 45 °C.

The IEC of the studied ionomeric membrane has a great influence on proton transfer via ionic channels/sites in the IPMC membrane, and helps in the evaluation of the working performance of the actuator. The IEC of the studied membrane was evaluated and found to be ca. 1.98 meq·g^−1^. The high IEC value of the IPMC membrane leads to an increase in the WU capacity, and allows more Pt particles to intensely fix on both sides of the surfaces of the membrane. The uniform loading and the presence of the high amounts of Pt particles present on the surface of membrane reduce the resistance of the IPMC and, hence, can lead to a better bending performance [26]. Figure 5 and Figure 6 describe the WL and WU capacities of the studied SPVC–PTA–Pt IPMC membrane, respectively, and were found to be ca. 36% at 6 V for 9 min, and ca. 57% at 45 °C for 10 h, respectively.

The PC of the SPVC–PTA–Pt membrane was found to be 1.6 mS·cm^−1^. A high value of PC facilitates the migration of excess H^+^ ions in their hydrated form, exhibiting good actuation capacity.

### 3.2. FTIR Analysis

**Figure 7:**
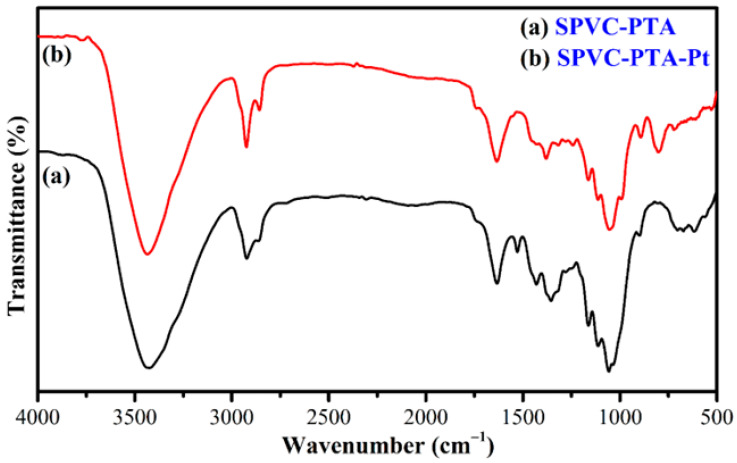
FTIR spectra of the (**a**) SPVC–PTA and (**b**) SPVC–PTA–Pt IPMC membranes.

Figure 7a,b represent the FTIR spectra of the studied SPVC–PTA and SPVC–PTA–Pt IPMC membranes, respectively. The spectrum of the SPVC–PTA polymer membrane shows a broad absorption peak at 3442 cm^−1^ due to the stretching mode of the –OH group. Meanwhile, the absorption peaks at 2918 and 2852 cm^−1^ were obtained due to the C-H stretching modes of the –CH_2_ and –CH_3_ groups, respectively, and the C–Cl stretching mode of PVC was observed at 642 cm^−1^ [1]. The characteristic absorption peaks at 1064 and 1034 cm^−1^, belonging to S=O and O=S=O bonds’ stretching for sulfonate groups attached to the polymeric chains, were also seen. The peaks at 1522 cm^−1^ correspond to C=C stretching vibration of the benzenoid ring. Nevertheless, an increase in the intensity of the adsorption peaks due to an increase in the degradation of the sulfonate group using the concentrated sulfuric acid was also seen. FTIR studies of the SPVC–PTA–Pt IPMC membrane revealed that a strong peak near 1639 cm^−1^ contributed to vibration of the phospho groups of the PTA parts in the polymer composite.

### 3.3. X-ray Diffraction (XRD) Analysis

**Figure 8:**
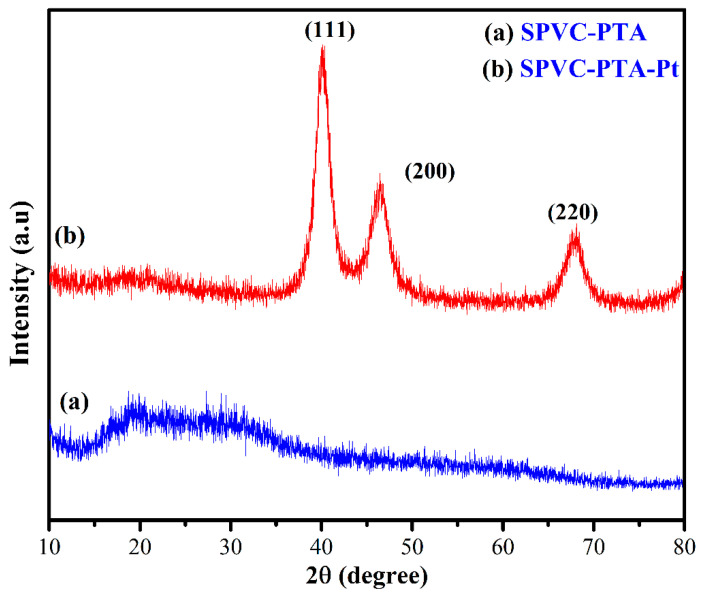
XRD spectra of the (**a**) SPVC–PTA and (**b**) SPVC–PTA–Pt IPMC membranes.

Figure 8a,b show the XRD patterns of the studied SPVC–PTA and SPVC–PTA–Pt IPMC membranes, respectively. Two different diffraction peaks are observed in Figure 8a at 2θ values of ca. 17.630 and ca. 24.380 for the SPVC–PTA membrane. Figure 8b presents the XRD pattern of the SPVC–PTA–Pt membrane, exhibiting the characteristic properties of crystalline Pt with a face-centered cubic (fcc) lattice. The peaks were characterized—first the (111) peak, followed by the (200) and (220) peaks—to 2θ values of ca. 39.450, 45.960, and 67.360, respectively. The relative sharpness of the observed peaks may be associated with an increase in the crystalline structures of the studied polymer membranes due to H bonding with the sulfonic groups, causing alignment of the polymer chains. Upon blending SPVC–PTA with Pt, the patterns of the XRD peaks of the prepared SPVC–PTA–Pt IPMC membranes revealed similar diffraction peaks for SPVC. These investigations of the XRD spectra exhibited an amorphous nature of the SPVC–PTA–Pt IPMC membrane [26,27].

### 3.4. Thermogravimetric Analysis (TGA) Study

**Figure 9:**
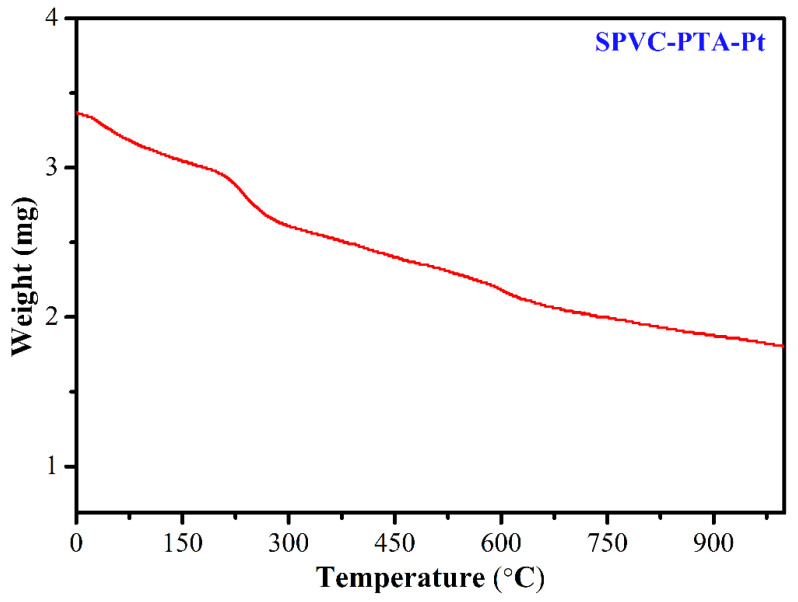
TGA curve of the SPVC–PTA–Pt IPMC membrane.

Figure 9 describes the TGA study of the prepared SPVC–PTA–Pt IPMC membrane, which shows the percentage weight loss as a function of temperature. This membrane exhibited a typical three-step pattern of degradation, which occurred near T = 100, 300, and 950 °C. In the first step, the maximum weight loss was found to be ca. 26% in the range of 50–100 °C due to the removal of H_2_O molecules bonded or absorbed to the sulfonic group, while in the second step, the loss was ca. 16% near 150–260 °C, due to the dissociation of olefinic chlorine (dehydrochlorination) [28]. The decrease in the percentage weight loss was due to a proportional increase in the molar concentration of sulfuric acid. The third weight loss observed during 450–650 °C and above 650 °C was attributed to the slow degradation of PVC. The slow and prolonged degradation could be due to multiple reasons, including sulfonation of the PVC, addition of phosphotungstic acid, and the Pt coating of the membrane, which collectively stabilized the polymer backbone. The TGA study reveals that the SPVC–PTA–Pt IPMC shows excellent (slow and prolonged) thermal stability.

### 3.5. SEM Study

**Figure 10:**
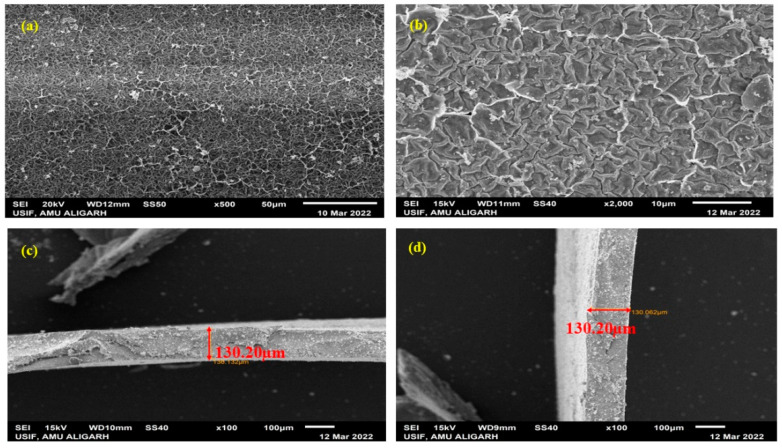
SEM images of the SPVC–PTA–Pt IPMC membrane.

Figure 10a–d show the surface and cross-sectional morphologies of the prepared SPVC–PTA–Pt IPMC membrane. Figure 10a,b, showing the surface morphology of the membrane at magnifications of 500× and 2000×, respectively, reveal the morphology of the self-assembled chains of the SPVC–PTA–Pt IPMC membrane. Meanwhile Figure 10c,d show the surface and cross-sectional views, respectively, at 100× magnification. The smooth texture at the membrane surface reflects the coating of the membrane surface with Pt metal, while the same images in the cross-sectional part show two boundaries: one is rough, showing the inner part of the IPMC membrane, which is not coated with Pt; while the other is smooth, related to the depth of the Pt coating of the membrane. The thickness of the Pt coating is ca. 30–35 μm, while the thickness of the whole IPMC membrane is ca. 200 μm. These images clearly indicate the successful coating of the SPVC–PTA membrane with the Pt metal.

### 3.6. EDX

**Figure 11:**
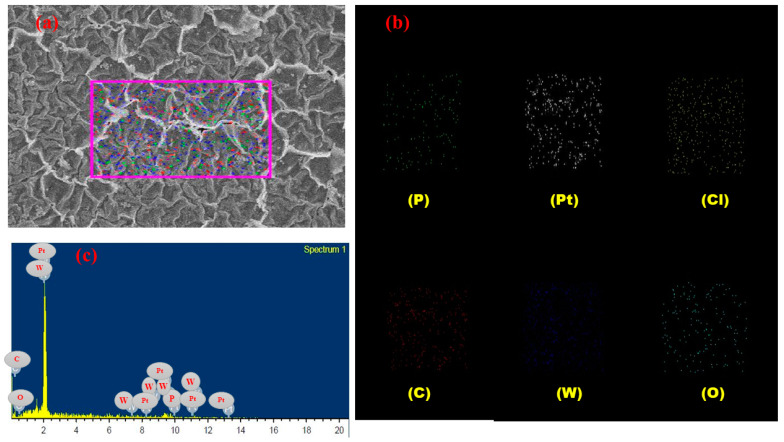
Represent the (**a**) surface of the IPMC membrane for EDX analysis (**b**) characteristic peaks for elements present in the membrane, and (**c**) distribution map for element in the IPMC membrane.

Figure 11 represents the EDX results, characteristic peaks, and distribution maps for each major element at the surface of the SPVC–PTA–Pt IPMC membrane. The EDX spectrum of the membrane surface exhibits the characteristic peaks of the elements present in the membrane, reflecting (qualitatively the amounts) of carbon (C), oxygen (O), phosphorus (P), chlorine (Cl), tungsten (W), and platinum (Pt) at the surface of the actuator. The Pt represents the highest peak, showing an excellent coating of the metal electrode at the surface of the membrane actuator.

### 3.7. Electromechanical Characterization

To characterize the actuation force behavior of the SPVC–PTA–Pt polymer actuator, a digital weighing scale was used. The tip of a cantilevered SPVC–PTA–Pt polymer actuator came into contact with the pan of the digital weighing scale and, after applying a controlled 4 V DC voltage, the different trials for force characterization were arranged with the SPVC–PTA–Pt polymer soft actuator, and the data were experimentally obtained, as shown in Table 2.

**Figure 12:**
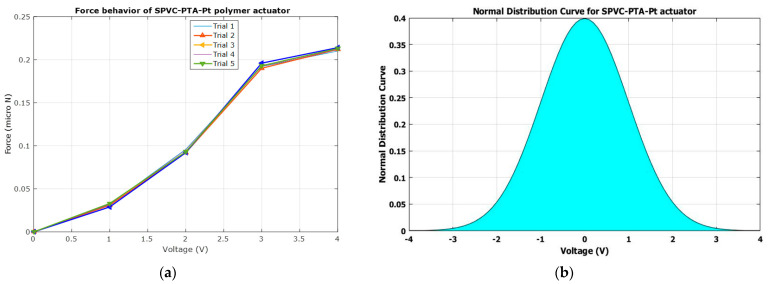
(**a**) The actuation force behavior of the SPVC–PTA–Pt bending actuator; (**b**) normal distribution behavior of the SPVC–PTA–Pt bending actuator.

It can be observed that the maximum force that can be attained by the SPVC–PTA–Pt polymer soft actuator is ca. 0.242 mN, as shown in Table 2 and Figure 12a. A normal distribution was also drawn for obtaining the SPVC–PTA–Pt polymer actuator, as shown in Figure 12b, which shows a sharp shape and good repeatability behavior of the force characteristics. It also shows that the normal distribution curve has zero values at maximum voltages on both the negative and positive sides. The repeatability of the polymer actuator was found to be ca. 91.29%.

## 4. Conclusions

This article presents the development of a novel SPVC–PTA–Pt-based IPMC soft actuator membrane. The structural, thermal, morphological, and electromechanical characterizations were performed using various techniques. The high PC (σ), high WU capacity, low WL, and high IEC under applied electric potential led to a good actuation performance (force and tip deflection/displacement) and repeatability with the SPVC–PTA–Pt IPMC membrane. Electromechanical results revealed that the developed IPMC membrane can be used for handling soft objects. Using a developed membrane, a micro-gripping system could be designed for soft robotic applications in the future.

## Figures and Tables

**Table 1:** Deflection data (experimentally obtained) of the SPVC–PTA–Pt polymer soft actuator.

Voltage			Deflection (mm)		
(V)	0.0	1.0	2.0	3.0	4.0
Trial 1	0	6.5	9.0	11.0	15.0
Trial 2	0	6.0	8.9	10.8	15.1
Trial 3	0	6.3	8.8	10.9	15.0
Trial 4	0	6.8	8.6	11.1	15.2
Trial 5	0	7.0	8.7	10.6	15.3

**Table 2:** Force data of the SPVC–PTA–Pt polymer soft actuator.

Voltage (V)	F_1_ (mN)	F_2_ (mN)	F_3_ (mN)	F_4_ (mN)	F_5_ (mN)	Average Force Value (F) in mN
0	0	0	0	0	0	0
1	0.032	0.031	0.029	0.032	0.03	0.0308
2	0.096	0.092	0.092	0.095	0.092	0.0934
3	0.192	0.19	0.196	0.192	0.19	0.192
4	0.202	0.212	0.232	0.222	0.242	0.222
Mean						0.107
Standard Deviation						0.087
Repeatability						91.29%

## Data Availability

Data are contained within the article.
